# Pioglitazone Is a Mild Carrier-Dependent Uncoupler of Oxidative Phosphorylation and a Modulator of Mitochondrial Permeability Transition

**DOI:** 10.3390/ph14101045

**Published:** 2021-10-14

**Authors:** Ekaterina S. Kharechkina, Anna B. Nikiforova, Konstantin N. Belosludtsev, Tatyana I. Rokitskaya, Yuri N. Antonenko, Alexey G. Kruglov

**Affiliations:** 1 Institute of Theoretical and Experimental Biophysics, Russian Academy of Sciences, Institutskaya 3, 142290 Pushchino, Russia; katya.kypri@gmail.com (E.S.K.); nikiforanna@yandex.ru (A.B.N.); bekonik@gmail.com (K.N.B.); 2 Mari State University, pl. Lenina 1, 424001 Yoshkar-Ola, Russia; 3 Belozersky Institute of Physico-Chemical Biology, Lomonosov Moscow State University, 119991 Moscow, Russia; rokitskaya@belozersky.msu.ru (T.I.R.); antonen@belozersky.msu.ru (Y.N.A.)

**Keywords:** permeability transition pore, unilamellar vesicles, adenine nucleotide translocase, uncoupling protein, ATP production

## Abstract

Pioglitazone (PIO) is an insulin-sensitizing antidiabetic drug, which normalizes glucose and lipid metabolism but may provoke heart and liver failure and chronic kidney diseases. Both therapeutic and adverse effects of PIO can be accomplished through mitochondrial targets. Here, we explored the capability of PIO to modulate the mitochondrial membrane potential (ΔΨ_m_) and the permeability transition pore (mPTP) opening in different models in vitro. ΔΨ_m_ was measured using tetraphenylphosphonium and the fluorescent dye rhodamine 123. The coupling of oxidative phosphorylation was estimated polarographically. The transport of ions and solutes across membranes was registered by potentiometric and spectral techniques. We found that PIO decreased ΔΨ_m_ in isolated mitochondria and intact thymocytes and the efficiency of ADP phosphorylation, particularly after the addition of Ca^2+^. The presence of the cytosolic fraction mitigated mitochondrial depolarization but made it sustained. Carboxyatractyloside diminished the PIO-dependent depolarization. PIO activated proton transport in deenergized mitochondria but not in artificial phospholipid vesicles. PIO had no effect on K^+^ and Ca^2+^ inward transport but drastically decreased the mitochondrial Ca^2+^-retention capacity and protective effects of adenine nucleotides against mPTP opening. Thus, PIO is a mild, partly ATP/ADP-translocase-dependent, uncoupler and a modulator of ATP production and mPTP sensitivity to Ca^2+^ and adenine nucleotides. These properties contribute to both therapeutic and adverse effects of PIO.

## 1. Introduction

PIO is a member of the thiazolidinedione class of insulin-sensitizing drugs, which are extensively used in the treatment of type 2 diabetes. Its impact on diabetes is linked primarily to the binding and activation of the peroxisome proliferator-activated receptor-γ, which regulates the expression of numerous insulin-responsive genes involved in the control of glucose and lipid metabolism [1]. The results of recent meta-analyses of population studies indicate that PIO efficiently decreases the blood pressure, the level of triglycerides, glycated hemoglobin, and blood glucose in fasting animals, as well as the risk of hypoglycemia [2,3,4]. Besides, due to its anti-inflammatory, antioxidant, and, perhaps, antibacterial and antifungal properties, PIO is now considered as a promising medicine for the treatment of a range of pathologic states, including Alzheimer’s disease [5], depressive disorder [6], non-alcoholic fatty liver disease [7], renal ischemia-reperfusion injury [8], *Klebsiella pneumoniae* infection [9], fibromyalgia-associated motor dysfunctions [10], respiratory infections (including Coronavirus disease 2019) [11,12], chronic obstructive pulmonary disease [13], cryptococcal meningitis [14], and ischemic outcomes induced by mild traumatic brain injury [15]. The intake of PIO is associated with a decrease in carotid intima-media thickness, a hallmark of atherosclerosis progression [2,16]. Although PIO decreases the probability of myocardial infarction and stroke in patients with clinical manifestations of cardiovascular disease, it does not reduce the all-cause mortality and increases the risk of heart failure [17,18]. Furthermore, PIO significantly raises the frequency of peripheral oedema [2], which may be due to fluid retention but not cardiac dysfunction [19]. What is more, PIO augments the probability of a newly developed chronic kidney disease [20]. Liver dysfunctions, including hepatitis, deregulation of the level of hepatic enzymes, and mixed hepatocellular-cholestatic liver injury, as well as liver failure with or without fatal outcomes, have been reported to be associated with the intake of PIO [21]. Besides, PIO significantly increases the risk of bladder cancer [22] but can display cytotoxic and cytostatic effects on several cancer cell lines [23,24,25,26].

Several mechanisms were proposed to explain the cardioprotective effect of PIO: anti-inflammatory effect [24,27], upregulation of mitochondrial antioxidant proteins [28,29], activation of mitochondrial АТР-sensitive K^+^ channel [30], induction of peroxisome proliferator-activated receptor gamma/peroxisome proliferator-activated receptor gamma coactivator 1-alpha signaling pathway [29], activation of pro-survival signaling phosphoinositide 3-kinases and P42/44 mitogen-activated protein kinases [31], cycloxygenase-2 and cytosolic phospholipase A2 [32], and inhibition of apoptosis [33]. The mechanisms of the adverse effects of PIO are unclear. According to a recent study, thiazolidinediones (including PIO) can induce toxicological molecular alterations via induction of cytochrome p450s that synthesize cardiotoxic 20-hydroxyeicosatetraenoic acid [34]. However, some evidence indicates that they are mitochondria-dependent and involve apoptosis, ferroptosis, autophagy, and mPTP opening [23,24,33,35,36,37].

One of the mitochondrial PIO targets is CDGSH iron-sulfur domain-containing protein 1 (mitoNEET) in the outer membrane [38]. MitoNEET transfers the 2Fe-2S cluster to cytosolic apoproteins, such as aconitase and apoferredoxin [39]. MitoNEET plays an important role in the regulation of iron homeostasis [40,41], ferroptosis [41], and mitochondrial biogenesis and network [35,42], oxidative capacity [43], reactive oxygen species production, and autophagy [35,40,44,45]. MitoNEET can be involved in glucose and lipid metabolism since the level of protein expression in pancreatic α- and β-cells regulates insulin secretion, glucose tolerance, and mitophagy [46], while in adipocytes it controls lipid uptake and storage [47,48]. It was shown that PIO stabilizes the 2Fe-2S cluster in mitoNEET [49] and prevents its transfer to apoproteins and iron transport to the mitochondria [50].

Another potential target for PIO in the mitochondria is glyceraldehyde 3-phosphate dehydrogenase (GAPDH), which is a proapoptotic regulator of mitochondrial membrane permeabilization [51]. It was shown that, at high concentrations (>100 µM), PIO inhibits the activity and decreases the expression of GAPDH [52].

The data on the direct effect of PIO on mitochondrial functions are limited. PIO was reported to decrease ΔΨ_m_ and uncouple the oxidative phosphorylation via the upregulation of the uncoupling protein 2 [43,44] and the production of reactive oxygen and nitrogen species [47]. Besides, it was found that PIO decreases state 3 mitochondrial respiration due to the inhibition and disassembly of complex I [23,53] and the inhibition of complex III [54,55]. Other studies did not confirm the uncoupling [54,56] and the inhibition of complexes by PIO [57]. In mitochondria isolated from hepatocytes and human skeletal muscles, PIO inhibited the ATP production [36,58], which may be connected with the stimulation of mPTP opening [36]. Other researchers reported that PIO does not affect the mPTP opening and ATP production [54].

Thus, it is unclear whether PIO is capable of directly modulating mitochondrial functions. At the same time, the structure of the PIO molecule allows reversible protonation (pKa 5.19) [59] and incorporation into the lipid bilayer (octanol-water partition coefficient ~2.3). These properties are inherent in conventional protonophores, which can penetrate through membranes both in neutral (protonated) and anionic forms [60]. Therefore, the aims of the present study were to assess the protonophoric and uncoupling effects of PIO in mitochondria and artificial lipid membranes and to clarify the effect of PIO on the mPTP opening in different models in vitro.

## 2. Results

### 2.1. PIO Acts as a Low-Efficient Uncoupler in Isolated Mitochondria

**Figure 1:**
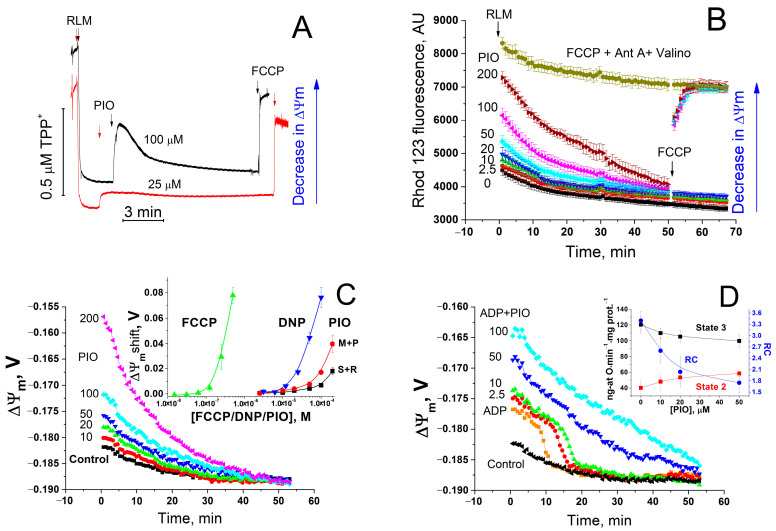
Effect of PIO on ΔΨ_m_ and the coupling of mitochondria. (**A**,**B**) Assessment of the effect of PIO on ΔΨ_m_ using TPP^+^ (**A**) and rhodamine 123 (Rhod 123, (**B**)). (**C**,**D**) Effect of PIO on ΔΨ_m_ in mitochondria in the resting state (**C**) and during the ADP phosphorylation (**D**). (**A**) Rat liver mitochondria (RLM) (1 mg prot./mL) were placed in a standard incubation medium supplemented with 5 mM K^+^-succinate, 1 mM EGTA, rotenone (2 µg/mL), and 1 µM TPP^+^. Arrows show the addition of 25 or 100 µM PIO and 500 nM FCCP. (**B**–**D**) A mitochondrial suspension (0.75 mg/mL) supplemented with respiratory substrates, 1 mM EGTA, and 330 nM rhodamine 123 was placed in wells with 1% dimethyl sulfoxide (DMSO) (vehicle control) or PIO at indicated concentrations (µM) (**B**–**D**), 500 nM FCCP, antimycin A (2.5 µg/mL), and valinomycin (25 ng/mL) (**B**), and 2 mM ADP (**D**) just before measurements. Everywhere, except the curve designated “M+P” (5 mM malate + 5 mM pyruvate), the respiratory substrate was 5 mM K^+^-succinate with the addition of rotenone (2 µg/mL). Numbers on curves are the means ± S.E.M. (*n* = 3) of one representative experiment of three identical experiments. (**B**) Where indicated, 500 nM FCCP was added to wells with 50, 100, and 200 µM PIO to calibrate the signal. Insert in panel (**C**) shows a shift in ΔΨ_m_ caused by FCCP, DNP, and PIO after 5 min of incubation. Insert in panel (**D**) shows the effect of PIO on the respiration rate in state 2 and state 3 and the respiratory control (RC) coefficient. In inserts, numbers on curves are the means ± S.E.M. of three independent experiments (*n* = 9 (**C**) and 3 (**D**)).

First, we examined the effect of PIO on ΔΨ_m_ and oxidative phosphorylation (Figure 1). As follows from the figure, PIO reduced the accumulation of rhodamine 123 in mitochondria (Figure 1B) and induced a release of accumulated tetraphenylphosphonium (TPP^+^) (Figure 1A), indicating a decrease in ΔΨ_m_. The determination of ΔΨ_m_ in the presence of PIO (Figure 1C,D) revealed a temporary dose-dependent decrease (up to 50 mV) in membrane potential with a subsequent slow return to control values (Figure 1C). PIO was a weaker proton gradient disrupter than carbonyl cyanide p-(trifluoromethoxy)phenyl-hydrazone (FCCP) and 2,4-dinitrophenol (DNP) (by two to three orders of magnitude and several times, respectively) (Figure 1C, insert). Besides, PIO strongly decreased ΔΨ_m_ in the presence of substrates of complex I compared to substrates of complex II. Even at a low concentration (2.5–20 µM), PIO noticeably reduced the efficiency of oxidative phosphorylation (Figure 1D and insert). It stimulated state 2 respiration and decreased the respiratory control coefficient (RC) and, though only slightly, the rate of state 3 respiration. Thus, PIO acts as a low-efficient protonophoric uncoupler, such as natural bile acids [61].

### 2.2. PIO Modulates the Sensitivity of the Permeability Transition Pore to Regulators

**Figure 2:**
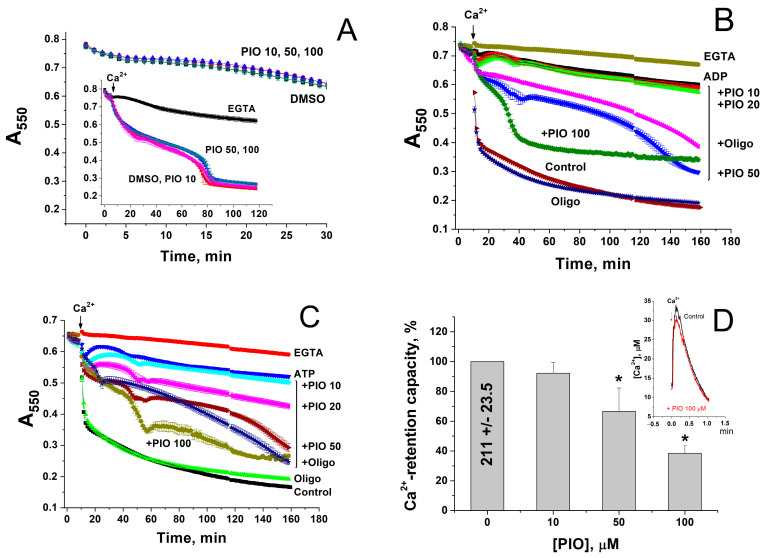
PIO increases mitochondrial sensitivity to Ca^2+^ and decreases the protective effect of mPTP inhibitors, ATP and ADP. (**A**–**C**) Ca^2+^-dependent mitochondrial swelling in the presence of PIO and ATP/ADP. (**D**) Effect of PIO on the Ca^2+^-retention capacity of mitochondria. (**A**–**C**) Mitochondrial suspension (0.75 mg prot./mL) supplemented with 5 mM K^+^-succinate (plus 2 µg/mL rotenone) and, where shown, 2 mM ADP (**B**) and 2 mM ATP (**C**) (pH 7.4) were placed in wells with indicated additions: 1 mM EGTA, 1% DMSO, 10–100 µM PIO, and 10 µg/mL oligomycin (Oligo). The arrow shows the addition of 50 µM Ca^2+^. Representative data of three similar experiments are shown. Points on traces are the means ± S.E.M. (*n* = 3). (**D**) Ca^2+^-retention capacity equal to 100% corresponds to 210.9 ± 23.45 nmol Ca^2+^/mg protein. Values in columns are means ± S.E.M. (*n* = 3) of three independent experiments. The asterisk shows the significant difference from the vehicle control (*p* < 0.05). The insert shows the kinetics of accumulation of 30 µM Ca^2+^ in the absence (Control) and in the presence of 100 µM PIO.

It was reported that PIO stimulates the opening of mPTP [36]. However, mPTP opening can disrupt the transmembrane proton gradient and uncouple the respiration and oxidative phosphorylation. Therefore, we explored the effect of PIO on the mPTP opening in the mitochondria in different experimental conditions. Figure 2 shows that PIO (10–100 µM) neither induced the ionic permeability of the inner mitochondrial membrane (IMM) in KCl-based medium (Figure 2A) nor markedly influenced the mPTP opening by a single addition of Ca^2+^ (Figure 2A, insert). At the same time, PIO negligibly affected the Ca^2+^ uptake rate (Figure 2D, insert) but drastically reduced the mitochondrial Ca^2+^ retention capacity (Figure 2D) (mitochondria were exposed to 30 µM pulses). Besides, PIO noticeably weakened the inhibition of the mPTP opening by added ADP (Figure 2B) and ATP (Figure 2C) (ADP and ATP are strong natural mPTP inhibitors, which can be present in the cytosol of living cells at millimolar concentrations). Thus, PIO does not induce the mPTP opening itself but can modulate the mitochondrial susceptibility to mPTP regulators, Ca^2+^ and ATP/ADP, under certain conditions.

### 2.3. PIO Decreases the Capability of Mitochondria to Phosphorylate ADP in the Presence of Ca^2+^

**Figure 3:**
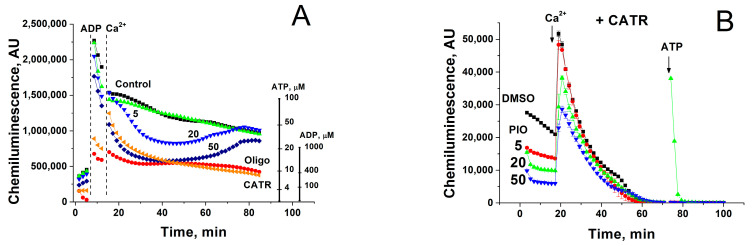
Effect of PIO on the capability of mitochondria to support ATP production in the presence of Ca^2+^ (**A**) and on the level of matrix ATP (**B**). Mitochondria (0.2 (**A**) and 0.5 mg prot./mL (**B**)) were placed in the standard incubation medium, which contained a 20% solution of a luciferin-luciferase reagent, 5 mM K^+^-succinate, 2 µg/mL of rotenone, 10 µM EGTA, and, where indicated, 5–50 µM PIO, 1 µM CATR, and 10 µg/mL oligomycin (Oligo). Vertical dashed lines (**A**) or arrows (**B**) show the addition of 500 µM ADP, 50 µM Ca^2+^, and 5 µM ATP standard (to calibrate the chemiluminescent signal). Representative data of three separate experiments are shown. Traces are the means for three wells.

Mitochondria within a living cell permanently exist in intermediate 3–4 metabolic state and are exposed to repeating Ca^2+^ pulses from endoplasmic/sarcoplasmic reticulum and the extracellular space, particularly in excitable tissues. We examined the effect of PIO on the capacity of mitochondria to maintain the level of endogenous ATP and ATP production after a single Ca^2+^ pulse (Figure 3). The release of ATP from intact mitochondria was traced using a 20% solution of a luciferin-luciferase reagent (without lysis buffer), which rapidly oxidizes external ATP and generates chemiluminescence. It should be noted that the luminescence produced by luciferase in the presence of ATP (left scale line) was 20 times more intensive than in the presence of ADP (right scale line) [62]. The relatively low but sustained luminescence in a mitochondrial suspension was interpreted as a continuous exchange of small quantities of ATP for ADP/AMP between the medium and mitochondria, since it was completely inhibited by oligomycin (Figure 3A). As it follows from the figure, 500 µM ADP caused a strong increase in the chemiluminescence, which comprised two constituents: (1) oligomycin-sensitive, dependent on the ATP generated by F_o_F_1_-ATPase, and (2) oligomycin-insensitive, dependent on ADP and ATP produced by adenylate kinase in the intermembrane space. PIO decreased the ADP-stimulated ATP release in a dose-dependent manner. Ca^2+^ further diminished the ATP production, which transiently became negligible at 50 µM PIO. Moreover, PIO reduced the level of matrix ATP, which can be released by the small Ca^2+^-activated mitochondrial carrier protein (SCaMC) in exchange for inorganic phosphate (Figure 3B) [63]. Hence, PIO strongly decreases the level of matrix ATP and reduces the capacity of the mitochondria for ATP production after a pulse of Ca^2+^.

### 2.4. Effect of PIO on the Permeability of the Inner Mitochondrial and Lecithin Liposomal Membranes to Protons

**Figure 4:**
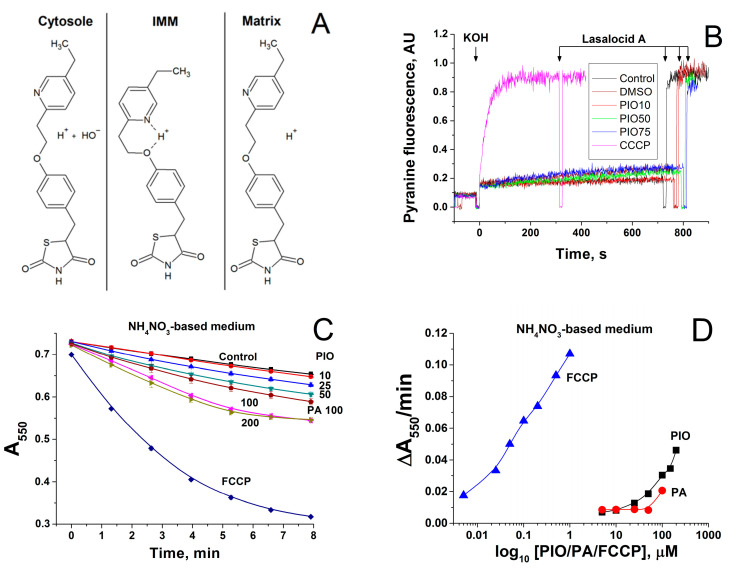
Mechanism of transmembrane proton transport by PIO. (**A**) Possible mechanism of proton transport by PIO across the IMM. (**B**) Comparison of CCCP- and PIO-mediated proton fluxes through liposomes loaded with the pH probe pyranine. The inner liposomal pH value was estimated from the pyranine fluorescence intensity measured at 505 nm upon excitation at 455 nm. Lasalocid A (1 µM) was added approximately at 800 s to equilibrate pH. PIO concentrations were 10 µM, red curve; 50 µM, green curve; 75 µM, blue curve. CCCP concentration was 1 µM (pink curve). Lipid concentration was 20 μg/mL, T = 15 °C. Other conditions: see Materials and Methods Section. The proton flux was initiated by an alkaline pH shift from рН 6 to рН 8, which was caused by the addition of the previously determined aliquot of KOH. In the presence of the protonophores, the pyranine fluorescence gradually increased, indicating the alignment of the pH values inside and outside liposomes due to proton transfer mediated by a protonophore. (**C**,**D**) Effect of PIO, palmitic acid, and FCCP on the swelling of deenergized mitochondria in NH_4_NO_3_-based medium. Mitochondria (0.75 mg prot./mL) were added to NH_4_NO_3_-based medium supplemented with rotenone (2.5 mg/mL), and 1 min later, the suspension was placed in the wells of a plate that contained PIO, sodium palmitate (PA), and FCCP at indicated concentrations. (**C**) Standard curves of mitochondrial swelling. (**D**) Initial rates of mitochondrial swelling during the first 3 min of incubation. Points are the means ± S.E.M. of three independent experiments (*n* = 9).

The molecular structure of PIO indicates that it may be reversibly protonated as conventional protonophores, such as FCCP or carbonyl cyanide m-chlorophenyl hydrazone (CCCP) (Figure 4A). Therefore, we studied the effect of PIO on the pH value in large unilamellar lecithin vesicles loaded with the fluorescent pH probe pyranine. Figure 4B shows the kinetics of dissipation of a pre-formed pH gradient on membranes of liposomes after the addition of PIO (red, green, and blue curves) or CCCP (pink curve). Black and brown curves show the control and vehicle control (15 µL DMSO), respectively. In contrast to the effect of CCCP, the addition of different concentrations of PIO (10 µM, red; 50 µM, green; 75 µM, blue) did not lead to an increase in the signal of pyranine (Figure 4B). It can be concluded that PIO is not able to induce proton transport in a pure lipid system.

We examined whether the effect of PIO on ΔΨ_m_ in mitochondria is connected with the activation of proton inward transport or with other reasons. Figure 4C shows that PIO accelerated the swelling of deenergized mitochondria in NH_4_NO_3_-based medium in a dose-dependent manner. This indicates that PIO, indeed, increased the permeability of the IMM to protons. PIO was a proton carrier three orders of magnitude weaker than FCCP but slightly more efficient than free palmitic acid (Figure 4D). Hence, PIO can transport protons in mitochondria but cannot do so in/out of liposomes.

### 2.5. Role of Mmitochondrial Carriers in PIO-Dependent Depolarization of Mitochondria

**Figure 5:**
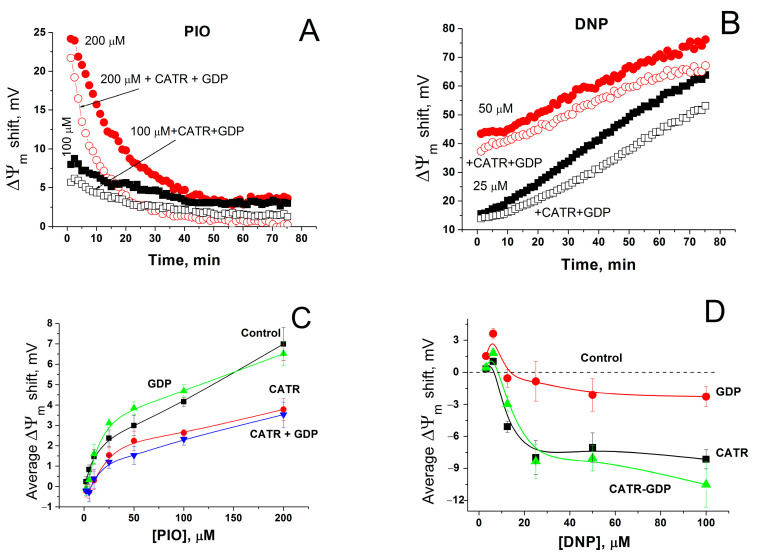
Effect of inhibitors of ANT and UCPs on the shift in ΔΨ_m_ caused by PIO and DNP. Incubation medium contained indicated concentrations of PIO and DNP, and, where shown, 1 mM GDP and 2 µM CATR. (**A**,**B**) Dynamics of ΔΨ_m_ changes (decrease) in relation to control (1% DMSO). The data of one representative experiment of three similar experiments are shown. Values on traces are the means for three wells. (**C**) Effect of GDP and CATR on the average PIO-dependent shift in ΔΨ_m_. Each point on the curves is an average ΔΨ_m_ shift defined as a mean ± S.E.M. for 60 points of experimental curves (such as in Panel (**A**)) for three individual experiments (*n* = 180). (**D**) Changes in the DNP-dependent ΔΨ_m_ shift caused by CATR and GDP in relation to DNP alone (Control).

Mitochondrial carriers are known to contribute to mitochondrial depolarization caused by fatty acids and other reversibly protonated compounds. The ADP/ATP carrier (ANT) facilitates the transport of fatty acid anions from the inner to the outer leaflet of the IMM and, therefore, reinforces the uncoupling of oxidative phosphorylation [64,65]. Uncoupler proteins 1–3 (UCPs) possess a similar activity [65,66]. We assessed the effect of the ANT and UCPs inhibitors carboxyatractyloside (CATR) and GDP on mitochondrial depolarization caused by PIO and conventional protonophores DNP and FCCP. Figure 5A shows that CATR plus GDP diminished the positive shift in ΔΨ_m_ (depolarization of the inner membrane) by PIO by 1–2 (100 µM PIO) and 3–5 mV, temporarily up to 10 mV (200 µM PIO), i.e., ~by 50%. The relative effect of DNP was less sensitive to CATR plus GDP: the decrease in the positive shift was up to 15 mV (15–20%) depending on the DNP concentration and incubation time (Figure 5B). In contrast, CATR and GDP had a minor effect on the FCCP-dependent depolarization (not shown). The analysis of the effects of CATR, GDP, and their combination on the average PIO-dependent positive shift in ΔΨ_m_ demonstrated that both CATR alone and its combination with GDP reduced the shift to a similar extent (Figure 5C). GDP alone did not attenuate the mitochondrial depolarization. Similar results were obtained with DNP (Figure 5D) (Figure 5D shows changes in DNP-dependent ΔΨ_m_ shift (Control, dashed line) caused by CATR and GDP). Thus, in liver mitochondria, ANT contributes to PIO- and DNP-induced mitochondrial uncoupling, while UCPs do not.

### 2.6. PIO Causes Mild Mitochondrial Depolarization in Intact Cells

**Figure 6:**
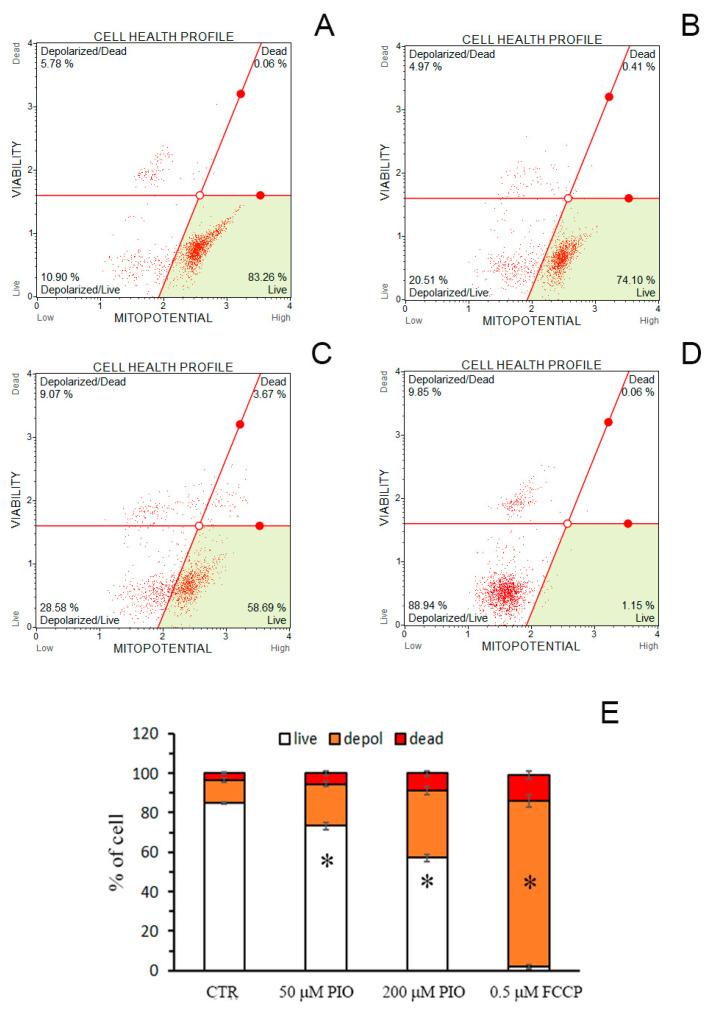
PIO decreases ΔΨ_m_ in isolated intact thymocytes. (**A**–**D**) Typical plots obtained in the absence of additions (**A**, Control), in the presence of 50 (**B**) and 200 µM PIO (**C**), and 500 nM FCCP (**D**). (**E**) The portion of depolarized cells in different experimental groups (means ± SEM, *n* = 3). * Statistically significant (*p* < 0.05) differences from control (without PIO).

We examined whether the effect of PIO on ΔΨ_m_ is preserved in intact cells. Figure 6 demonstrates that 30-min incubation of cells with 50 (Figure 6B,E) and 200 µM PIO (Figure 6C,E) significantly increased the portion of isolated thymocytes with depolarized mitochondria in comparison with the control (Figure 6A,E). However, the effect was substantially less pronounced than the effect of 500 nM FCCP (Figure 6D,E). Thus, PIO can act as a mild depolarizing agent in mitochondria of intact cells.

### 2.7. Effect of PIO on ΔΨ_m_ in Isolated Mitochondria and Mitochondria in Nuclei-Free Liver Homogenate

**Figure 7:**
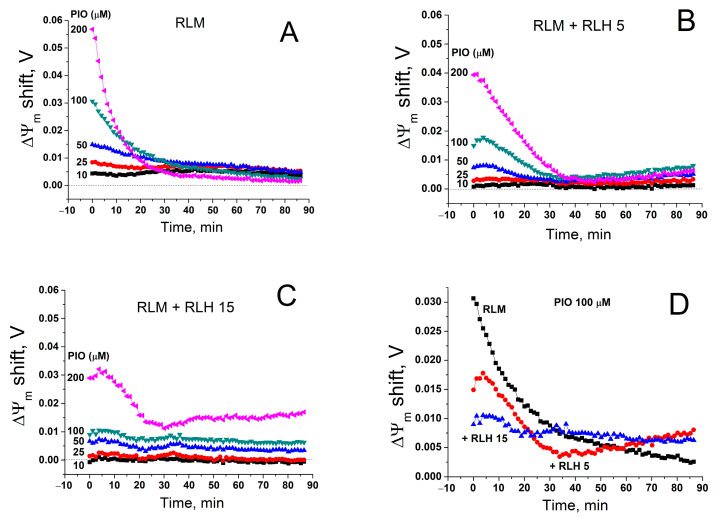
Effect of a cytosolic fraction (RLH) on PIO-dependent changes in ΔΨ_m_. Just before measurements, mitochondria alone (RLM) (0.75 mg prot./mL) (**A**,**D**) or in combination with 5 (**B**,**D**) and 15 mg prot./mL (**C**,**D**) of RLH were added to a standard incubation medium supplemented with 5 mM K^+^-succinate, 1 mM EGTA, rotenone (2.5 µg/mL), and 330 nM rhodamine 123, and then transferred to the wells of a 96-well plate, which contained 1% DMSO (Control), 10–200 µM PIO, or 500 nM FCCP, antimycin A (2.5 µg/mL), and valinomycin (25 ng/mL). Traces are the differences in ΔΨ_m_ between PIO- and DMSO-containing samples in one representative experiment of three similar experiments.

In isolated mitochondria, PIO-dependent depolarization was transient (see Figure 1, Figure 4 and Figure 5): the higher the PIO concentration was, the higher the rate of the loss of the effect. Since PIO is poorly soluble in water, one can assume that, at high concentrations, PIO forms suspension, which decreases the effective concentration of PIO and deteriorates its protonophoric effect. However, the cytosol of living cells comprises constituents that increase solubility and facilitate the transport of poorly soluble compounds [67,68]. Therefore, we compared the rate of restoration of ΔΨ_m_ in the presence of PIO in a standard mitochondrial suspension and a suspension that contained mitochondria- and nuclei-free rat liver homogenate (RLH) diluted to a final concentration of 5 (Figure 7B,D) and 15 mg prot./mL (Figure 7C,D).

As it follows from Figure 7, RLH diminished the initial PIO-dependent positive shift in ΔΨ_m_ (deteriorated the maximum depolarization) in a dose-dependent manner. Simultaneously, the depolarization induced by PIO at high concentrations became more sustainable (Figure 6C,D). Hence, cytosolic components, presumably, did not increase the solubility and the effective concentration of PIO but rather acted as a PIO buffer.

## 3. Discussion

The study confirmed that PIO can act as an uncoupler of oxidative phosphorylation, though much less efficient than FCCP (Figure 1 and Figure 5) and 2,4-dinitrophenol [69], but equally or more efficient than fatty (Figure 4) and bile acids [61,70]. However, despite the octanol-water partition coefficient of 2.3 and the pKa of 5.19 [59], PIO per se cannot operate either as a classical protonophore (Figure 4) or as a K^+^ ionophore (Figure 2) in mitochondrial and artificial lipid membranes. Nevertheless, PIO-dependent mitochondrial depolarization is associated with the activation of inward proton transport (Figure 4C,D).

It was established three decades ago that ANT can translocate fatty acid anions across the IMM, which strengthens the uncoupling effect of exogenous and endogenous fatty acids [64,71]. Substrates and inhibitors of ANT suppress the protonophoric effect of fatty acids [65,72]. UCPs, a family of proteins structurally related to ANT, can also mediate the fatty acid-dependent depolarization of the IMM and mitochondrial uncoupling [73]. According to recent findings, UCP2 possesses the activity of the fatty acid flippase, which is essential for proton conductance [66]. GDP interferes with the binding of fatty acids to UCP1 and 2 and suppresses proton currents [73]. Mitochondrial uncoupling by DNP is known to be partially dependent on ANT, while the effect of FCCP is carrier-independent [64]. Novel data indicate that both recombinant ANT1 and UCP1-3 considerably increase the protonophoric effect of DNP in planar bilayer lipid membranes and that arginine 79 of ANT1 is essential for DNP binding and translocation [65]. Here, we demonstrated that, in liver mitochondria, ANT considerably contributes to PIO-dependent mitochondrial depolarization and uncoupling (Figure 5). The role of UCP proteins demands further clarification since neither PIO- nor DNP-dependent depolarization was sensitive to the UCP inhibitor in liver mitochondria, which may be due to the low level of the carriers but not to an inability to transport PIO per se [65]. Thus, PIO-dependent mitochondrial depolarization and uncoupling is ANT-mediated, at least partially.

The interaction of PIO with ANT, an important regulator of mPTP, can explain the slight inhibition of state 3 respiration (Figure 1), the reduction of the mitochondrial Ca^2+^-retention capacity, and the cancellation of the suppression of mPTP by adenine nucleotides (Figure 2). Besides, by activating the inward proton transport, PIO can decrease the pH value in the mitochondrial matrix, which promotes the mPTP opening [74]. Another mechanism for facilitation of mPTP opening may be connected to the depletion of matrix ATP (Figure 3) via the Ca^2+^-activated SCaMC-mediated pathway [62,63,75].

A short duration of the PIO-dependent depolarization of isolated mitochondria in suspension (Figure 1, Figure 4 and Figure 5) is, presumably, connected with poor solubility in water (4–6.5 µg/mL or 11–16.5 µM) [67,76], but not with the glutathione-s-transferase-dependent inactivation of PIO, as it was shown for other uncouplers [77], since GSH had a negligible effect on the depolarization (not shown).

In living cells, however, the effect of PIO could be long-lasting. Indeed, the cytoplasm contains various compounds capable of acting as co-solvents and increasing the solubility and free concentration of PIO tens and hundreds of times [67]. Alternatively, multiple cytosolic proteins and systems facilitate the storage and transport of poorly soluble compounds [68]. The data obtained are in favor of the second mechanism, since the cytosolic fraction decreased the strength but increased the duration of PIO-dependent mitochondrial depolarization (Figure 7), i.e., acted as a PIO buffer. Thus, one can expect that PIO-dependent mitochondrial depolarization will be mild (Figure 6) but sustained or even permanent in prolonged treatment in situ or therapy and in vivo.

**Figure 8:**
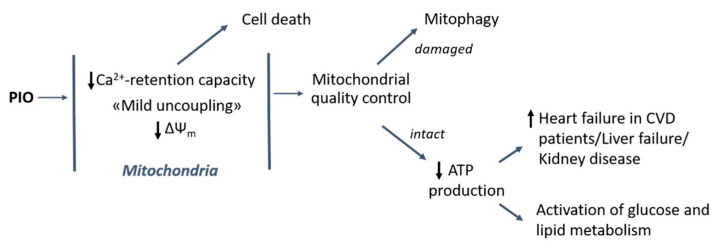
Contribution of uncoupling and mPTP-modulating effects of PIO to its healing and harmful action in the cell and the organism. CVD: cardiovascular disease.

The data obtained can help one explain some of the adverse effects of PIO in various organs and systems (Figure 8). The increased risk of heart failure in patients with cardiovascular disease [17] may be connected with the effect of PIO on the efficiency of ATP production under conditions of repeating Ca^2+^ pulses (Figure 3). In addition, the slow release of fluid, peripheral oedema, development of chronic kidney disease, and liver dysfunction promoted by PIO [2,19,20,21] can be attributed to insufficient production of ATP for ion pumps and exchangers in the plasma membrane. Moreover, the reduction of the mitochondrial Ca^2+^-retention capacity and the cancellation of the suppression of mPTP by adenine nucleotides (Figure 2) can be a reason for the toxicity of PIO in several cell lines [23,24,25,26]. At the same time, the reduction of the risk of myocardial infarction and stroke in patients with clinical manifestation of cardiovascular disease might be connected with long-term effects of PIO, such as the regulation of autophagy and mitochondrial quality control (Figure 8) [33]. Further, mild mitochondrial uncoupling and the activation of glucose and fat metabolism should contribute to the antidiabetic and anti-atherosclerotic action of PIO [2,16,78]. Indeed, another uncoupler, DNP, was successfully applied for the correction of glucose and lipid metabolism in animals and humans [79,80] and is now considered as a promising medicine for the treatment of a range of pathologic states [69].

## 4. Materials and Methods

### 4.1. Materials

ATP Kit SL (144-041) was purchased from BioThema AB (Haninge, Sweden). ADP (sodium salt) (A2754), ATP (disodium salt hydrate) (A7699), antimycin A (A8674), bovine serum albumin (BSA) (A7030), FCCP (C2920), CATR (C4992), DMSO (276855), DNP (D198501), GDP (sodium salt) (G7127), 4-(2-hydroxyethyl)piperazine-1-ethanesulfonic acid (HEPES) (H3375), palmitic acid (P0500), PIO (hydrochloride) (E6910), rhodamine 123 (R8004), rotenone (R8875), sucrose (S7903), succinate (S3674), TPP^+^ (chloride) (218790), Trizma Base (93352), and valinomycin (94675) were purchased from the Sigma-Aldrich Corporation (St. Louis, MO, USA). Ethylene glycol-bis(2-aminoethylether)-N,N,N′,N′-tetraacetic acid (EGTA) (A0878,0025) was from PanReac ApppliChem (Darmstadt, Germany). Other chemicals were of analytical grade and were purchased from local suppliers (Moscow, Russia).

### 4.2. Isolation of Rat Liver Mitochondria and Preparation of the Cytosolic Fraction of Liver Homogenate

All manipulations with animals before the isolation of the liver were performed in accordance with the Helsinki Declaration of 1975 (revised in 1983), national requirements for the care and use of laboratory animals, and protocol 9/2020 of 17.02.2020 approved by the Commission on Biological Safety and Bioethics at the ITEB RAS.

Rat liver mitochondria were isolated by a standard differential centrifugation procedure [81]. Adult male Wistar rats were killed by cutting the neck after anesthesia with CO_2_. The homogenization medium contained 220 mM mannitol, 70 mM sucrose, 10 mM HEPES (pH adjusted to 7.4 with Trizma Base), 1 mM EGTA, and 0.05% BSA. The pellet was washed three times with a medium devoid of EGTA and BSA. Final pellets were resuspended in this medium to yield 60–70 mg protein/mL. Measurements were performed at 37 °C in KCl-based medium (125 mM KCl, 20 mM sucrose, 10 mM HEPES (pH adjusted to 7.3 with Trizma Base), 2 mM KH_2_PO_4_, 2 mM MgCl_2_, and 10 µM EGTA) supplemented with 5 mM potassium succinate and rotenone (2 µg/mL), unless otherwise indicated. Other experimental details are provided in figures and figure legends. The intactness of isolated mitochondria was assessed as described previously [82].

The cytosolic fraction of liver homogenate (mitochondria- and nuclei-free RLH) was prepared as follows. The liver (1 g) was homogenized in the standard homogenization medium devoid of BSA. The homogenate was centrifuged 700 g × 15 min and 15,000 g × 20 min. Pellets were discarded at each step. The resulting RLH was kept on ice until use.

The total protein in mitochondrial and cytosolic fractions was determined by the Biuret method using BSA as a standard [83].

### 4.3. Measurements of the Oxygen Consumption Rate

Mitochondrial respiration was measured using an oxygen Clark-type electrode in a temperature-controlled electrode chamber connected to a computerized recording system, Record 4 (Institute of Theoretical and Experimental Biophysics, Russian Academy of Sciences (ITEB-RAS), Russia). Mitochondria (1 mg/mL) were incubated at 25 °C in standard medium supplemented with 5 mM succinate in the presence of rotenone (2 μg/mL). In order to assess state 3 and state 4 respiration rates, 200–500 μM ADP was added to mitochondria respiring in the presence of substrates (state 2). The RC coefficient was defined as the ratio of the respiration rate in state 3 to the rate in state 4.

### 4.4. Measurements of ΔΨ_m_ in Isolated Mitochondria

ΔΨ_m_ across the IMM was measured using the ΔΨ_m_-sensitive fluorescent dye rhodamine 123 and a plate reader Infinite 200, Tecan (Grödig, Austria). Standard incubation medium contained respiratory substrates, 1 mM EGTA, 330 nM rhodamine 123, and, where indicated, 2 mM ADP, 1 mM GDP, 2 µM CATR, and PIO, FCCP, and RLH at different concentrations. In order to calibrate the fluorescent signal, each experimental series contained samples with a cocktail of respiratory inhibitors and ionophores, which disrupt ionic gradients across the IMM (500 nM FCCP, antimycin A (2.5 μg/mL), and valinomycin (25 ng/mL)) [84]. ΔΨ_m_ was calculated using the Nernst equation assuming that: (1) the matrix volume is equal to 1 µL/mg protein (liver mitochondria), (2) the fluorescence of rhodamine 123 is directly proportional to the concentration in solution and is totally quenched upon the accumulation in mitochondria, and (3) initial fluorescence in samples with a cocktail of respiratory inhibitors and ionophores corresponds to 300 nM rhodamine 123.

Alternatively, ΔΨ_m_ was assessed using a TPP^+^-selective electrode (Niko Analyt, Russia) connected to a computerized recording system, Record 4 (ITEB RAS). The electrode was calibrated with known amounts of TPP^+^ at the beginning of each experimental series.

### 4.5. Measurements of ΔΨ_m_ in Isolated Thymocytes

Thymocytes were isolated from two thymuses of male Wistar rats (90–110 g) in accordance with a known method [85]. Medium containing 145 mM NaCl, 5.6 mM KCl, 10 mM glucose, and 8 mM Mops-KOH (pH 7.4) was used to isolate, wash, suspend, and incubate the cells. In control samples, the cell survival was at least 90%. All experiments with cells were carried out for 3 h; in this case, the cells retained similar viability and mitochondrial potential, as assessed by flow cytometry using a Muse Cell Analyzer (Merck Millipore, Burlington, MA, USA).

The mitochondrial potential was assessed by using a Muse MitoPotential Kit (MCH100110, Merck Millipore, Burlington, MA, USA) in order to determine the percentages of cells exhibiting a change in mitochondrial polarization. All assays were performed strictly according to the manufacturer’s protocols.

### 4.6. Recording of the Permeabilization of Mitochondrial Membranes

The permeabilization of mitochondrial membranes for solutes was assessed by high-amplitude mitochondrial swelling. Mitochondrial swelling (a decrease in A550) was recorded using an Infinite 200 plate reader, Tecan, Austria, and 96-well plates. Other details are provided in figures and figure legends.

### 4.7. Ca^2+^-Retention Capacity of Mitochondria

Mitochondrial Ca^2+^ uptake and release were recorded in a temperature-controlled electrode chamber using a Ca^2+^-electrode connected to the computerized recording system, Record 4. The Ca^2+^-retention capacity was defined as the amount of Ca^2+^ mitochondria taken up in small pulses before the Ca^2+^ release. Mitochondria (1 mg/mL) were added to incubation medium (25 °C) supplemented with 5 mM K^+^-succinate plus 2 µg/mL rotenone. Other experimental details are provided in figure legends.

### 4.8. Measurement of ATP in Mitochondrial Suspension

The ATP content in a suspension of intact mitochondria was determined using an ATP Biomass Kit HS in accordance with the manufacturer’s instructions. Incubation medium contained a 20% luciferin-luciferase reagent, 5 mM K^+^-succinate, 2 µg/mL of rotenone, 10 µM EGTA, and 0.2 mg of mitochondrial protein/mL. Other experimental details are provided in figures and figure legends. The chemiluminescent signal was calibrated by additions of ATP standards and known amounts of ADP.

### 4.9. Assessment of the Protonophoric Properties of PIO Using Pyranine-Loaded Vesicles

The luminal pH of liposomes was assayed with pyranine by a slightly modified procedure of [86]. To prepare pyranine-loaded liposomes, a lipid (2 mg POPC, 1 mg POPG, and 1 mg cholesterol) in a chloroform suspension was dried in a round-bottom flask under a stream of nitrogen. The lipid was then resuspended in a buffer (100 mM KCl, 20 mM MES, 20 mM MOPS, 20 mM Tricine titrated with KOH to pH 6.0) containing 0.5 mM pyranine. The suspension was vortexed and then freeze-thawed three times. Unilamellar liposomes were prepared by extrusion through 0.1 µm pore size Nucleopore polycarbonate membranes using an Avanti Mini-Extruder. The unbound pyranine was then removed by passage through a Sephadex G-50 coarse column equilibrated with the same buffer solution. To measure the rate of pH dissipation in liposomes with luminal pH 6.0, liposomes were diluted in a solution buffered to pH 8 and supplemented with 2 mM p-xylene-bis-pyridinium bromide to suppress the fluorescence of leaked pyranine. The inner liposomal pH was estimated from the pyranine fluorescence intensity measured at 505 nm upon excitation at 455 nm with a Panorama Fluorat 02 spectrofluorometer [87]. At the end of each recording, 1 µM lasalocid A was added to dissipate the remaining pH gradient. To prevent the formation of H^+^-diffusion potential, the experiments were carried out in the presence of 10 nM valinomycin.

### 4.10. Assessment of PIO-Dependent Proton Transport in Mitochondria

The rate of proton transport was assessed by the PIO-dependent osmotic swelling of deenergized mitochondria in NH_4_NO_3_-based medium (135 mM NH_4_NO_3_, 0.5 mM EGTA, and 10 mM HEPES-KOH (pH 7.0)) [88]. Mitochondrial swelling was defined as a decrease in A_550_ using a plate reader (Infinite 200 Tecan, Austria) and 96-well plates. Other details are provided in figures and figure legends.

### 4.11. Statistical Analysis

The data shown represent the means ± standard error of means (S.E.M.) or are the means of at least three experiments. Statistical probability (*p*) values were derived by the Student’s t-test.

## 5. Conclusions

Thus, here, we demonstrated that PIO can behave as a carrier-dependent uncoupler, a regulator of the efficiency of ATP production, and a modulator of the mPTP sensitivity to Ca^2+^ and adenine nucleotides. These properties contribute to both therapeutic and adverse effects of PIO in cells and the organism.

## Data Availability

Data is contained within the article.
